# Visible/Near Infrared Spectroscopy and Chemometrics for the Prediction of Trace Element (Fe and Zn) Levels in Rice Leaf

**DOI:** 10.3390/s130201872

**Published:** 2013-02-01

**Authors:** Yongni Shao, Yong He

**Affiliations:** College of Biosystems Engineering and Food Science, Zhejiang University, Hangzhou 310058, China; E-Mail: ynshao@zju.edu.cn

**Keywords:** Vis/NIR spectroscopy, rice, traces elements, independent component analysis (ICA), least squares-support vector machine (LS-SVM)

## Abstract

Two sensitive wavelength (SW) selection methods combined with visible/near infrared (Vis/NIR) spectroscopy were investigated to determine the levels of some trace elements (Fe, Zn) in rice leaf. A total of 90 samples were prepared for the calibration (n = 70) and validation (n = 20) sets. Calibration models using SWs selected by LVA and ICA were developed and nonlinear regression of a least squares-support vector machine (LS-SVM) was built. In the nonlinear models, six SWs selected by ICA can provide the optimal ICA-LS-SVM model when compared with LV-LS-SVM. The coefficients of determination (R^2^), root mean square error of prediction (RMSEP) and bias by ICA-LS-SVM were 0.6189, 20.6510 ppm and −12.1549 ppm, respectively, for Fe, and 0.6731, 5.5919 ppm and 1.5232 ppm, respectively, for Zn. The overall results indicated that ICA was a powerful way for the selection of SWs, and Vis/NIR spectroscopy combined with ICA-LS-SVM was very efficient in terms of accurate determination of trace elements in rice leaf.

## 1. Introduction

Recently, variable selection or uninformative variable elimination has attracted more and more attention for the development of multi-component calibrations using spectroscopic techniques. The recently developed methods for variable selection include generalized simulated annealing [1], genetic algorithm [2], correlation coefficients and B-matrix coefficients [3], latent variables analysis (LVA) [4], x-loading weights [5], uninformative variable elimination [6], regression coefficient analysis (RCA) [7,8], independent component analysis (ICA) [9,10] and so on. Among these methods, ICA has recently attracted much attention and has been successfully used in many fields, e.g., medical signal analysis, image processing, dimension reduction, fault detection and near-infrared spectral data analysis [11–15].

Various calibration methods have been used to relate near-infrared spectra (NIRS) with measured properties of materials. Principal components regression (PCR), partial least squares (PLS), multiple linear regression (MLR) and artificial neural networks (ANN) are the most used multivariate calibration techniques for NIRS [16–19]. PLS is usually considered for a large number of applications in fruit and juice analysis and is widely used in multivariate calibration because it takes advantage of the correlation relationships that already exist between the spectral data and the constituent concentrations. However PLS is based on linear models and unsatisfactory results may occur when non-linearity is present [20,21].

The least-squares support vector machine (LS-SVM) can handle the linear and nonlinear relationships between the spectra and response chemical constituents [22,23], therefore, a new combination of ICA with LS-SVM was proposed as a nonlinear calibration model for quantitative analysis using spectroscopic techniques. The performance of ICA-LS-SVM was evaluated by a case study to determine the trace elements in rice, with the purpose of developing a fast and accurate nonlinear model using fewer selected variables for the determination of the trace elements in rice.

The objective of this study were (1) to investigate the feasibility of using Vis/NIRS to predict trace elements such as Fe and Zn in rice leaf; (2) to compare the performance of ICA and the newly proposed ICA-LS-SVM model, variable selection methods (PCA, LVA and ICA) to predict the trace elements in rice.

## 2. Materials and Methods

### 2.1. Experimental Design

The experimental samples in this study were 15 basins of rice, which were planted in conditioned soil with three nitrogen levels: 0, 120, and 240 kg/ha. To avoid accidental damage to the basins or samples, a duplicate set of basins was prepared, so there were 30 basins in total. For each nitrogen level, there were 10 basins, including the additional basins. Each basin's inner diameter and height were 30 and 45 cm, respectively. Each basin contained 10 kg soil and four rice plants. The basins were placed in a slotted field using the surrounding soil for backfill, and they were placed along the line from north to south. The soil used in this experiment was from the 20 to 40 cm depth of the experimental field.

### 2.2. Data Acquisition and Preprocessing

Three leaf samples from each of 15 basins were selected for spectral measurement. Samples were also selected from another 15 replicate basins, so a total of 90 samples were obtained. The measurements were made at the booting stages. All 90 leaf samples reflectance measurements were made using a portable Spectroradiometer (FieldSpec Vis/NIR, Analytical Spectral Device, Boulder, CO, USA), with a sensitivity range from 325 to 1,075 nm. The instrument uses a sensitive 512-element, photo-diode array spectroradiometer, with a resolution of 3.5 nm. The scan number for each spectrum was set to 10 at the same position, and for each sample, three reflection spectra were taken, thus a total of 30 data points were properly stored for later analysis. To achieve the relative reflectance measurements, the white reference (a white panel purchased with the spectroradiometer used as white reference) was collected before scanning samples until a nice, clean, 100% reference line was obtained. All leaves were randomly divided into two sets, one was used as a calibration set (n = 70) and the remaining samples as a validation set (n = 20). In order to compare the performance of different calibration models, the samples in the calibration and validation sets were kept the same for all the models.

### 2.3. Trace Elements (Fe, Zn) Measurement

In the study, we used the national standard method to measure the trace elements Fe and Zn [24]. First, HNO_3_, HClO_4_, and distilled water were diluted and adjusted to the required concentration solution. Rice leaf samples were finely ground and then passed through a 20 mesh sieve to obtain very fine particles. An air-dried, ground and sieved sample (2.0 g) was placed in an Erlenmeyer flask and the extracting solution (20 mL) was added. Then it was placed on a magnetic stirrer and the mixture was stirred for 20 minutes. The resulting solution was filtered through a filter paper into a 50 mL polypropylene vial and diluted to 50 mL with the extracting solution. After that, a Perkin-Elmer Analyst™800 atomic absorption spectrometer (PerkinElmer, Inc., Shelton, CT, USA) was used to measure the signal strength of the elements Fe and Zn in each Erlenmeyer flask, and the results were shown using the software package of the instrument. After calculation, the Fe content was from 39.951 ppm to 134.254 ppm, and Zn content was from 9.085 ppm to 49.927 ppm in all 90 samples. Table 1 shows the statistic values of Fe and Zn contents in calibration and validation sets.

### 2.4. Data Pretreatment

Due to the potential system imperfections, obvious scattering noises could be observed at the beginning and end of the spectral data. Thus, the first and last 75 wavelength data points were eliminated to improve the measurement accuracy, *i.e.*, all visible and NIR spectroscopy analyses were based on a 400–1,000 nm scan. The above spectral data preprocessing was finished in ViewSpec Pro V4.02 (Analytical Spectral Device, Inc.). After that, the spectral data was preprocessed using Savitzky-Golay smoothing with a window width of 7 (3-1-3) points [25]. The data preprocessing was implemented by the software Unscrambler V 9.6 (Camo Process AS, Oslo, Norway).

### 2.5. Principal Components Analysis (PCA)

Reducing the number of inputs to the LS-SVM can reduce training time. Furthermore, it can also reduce repetition and redundancy of the input spectra data. PCA is a method of data reduction that constructs new uncorrelated variables, known as principal components (PCs). They account for as much information as possible for the variability of the original variables, which are then used as the inputs of network. In addition, PCs can also eliminate noises and random errors in the original data. The equation of PCA could be described as follows:

(1) $$X=TP^{-1}+E$$

where X is a N × K data matrix, T is a N × A score vector matrix, P is a K × A loading vector matrix, E is a N × K residual matrix, N is the number of samples, K is the number of spectral variables, and A is the number of PCs.

### 2.6. Partial Least Squares Analysis

In the development of PLS model, calibration models were built between the spectra and the content of trace element (Fe and Zn), full cross-validation was used to evaluate the quality and to prevent over-fitting of calibration models. Latent variables (LVs) can be used to reduce the dimensionality of data, and the optimal number of LVs was determined by the lowest value of predicted residual error sum of squares (PRESS). The prediction performance was evaluated by the coefficients of determination (R^2^) and root mean square error of calibration (RMSEC) or prediction (RMSEP), and bias. The ideal model should have higher r value, lower RMSEC, RMSEP and bias. The RMSEP and bias could be calculated via:

(2) $$\text{RMSEC}(\text{RMSEP})=\sqrt{\frac{1}{I_{p}}\sum_{i=1}^{I_{p}}{\left({\hat{y}}_{i}-y_{i}\right)}^{2}}$$

(3) $$\text{bias}=\frac{1}{I_{p}}\sum_{i=1}^{I_{p}}\left({\hat{y}}_{i}-y_{i}\right)$$

where *ŷ_i_* is the predicted value of each sample in prediction set (RMSEP), *y_i_* is the measured value of the each sample, and *I_p_* is the sample number in the prediction set.

### 2.7. Independent Component Analysis

Independent component analysis is a well-established statistical signal processing technique that aims to decompose a set of multivariate signals into a base of statistically independent components with the minimal loss of information content. The independent components are latent variables, meaning that they cannot be directly observed, and the independent component must have non-Gaussian distributions. A brief explanation of noise-free ICA model could be expressed by the following equation:

(4) x=As

where x denotes the recorded data matrix, s and A represent the independent components and the coefficient matrix, respectively. The ICs were obtained by a high-order statistic, which is a much stronger condition than orthogonality. This goal is equivalent to find a separating matrix W that satisfies:

(5) $$\hat{s}=\text{Wx}$$

where *ŝ* is the estimation of s. The separating matrix W can be trained as the weight matrix of a two-layer feed-forward neural network in which x is input and *ŝ* is output.

There are lots of algorithms for performing ICA [26]. Among these algorithms, the fast fixed-point algorithm (FastICA), which was developed by Hyvarinen and Oja [27], is highly efficient for performing the estimation of ICA. FastICA was chosen for ICA and carried out in Matlab 7.0 (The Math Works, Natick, MA, USA).

### 2.8. Least Squares-Support Vector Machine

Least squares-support vector machine can work with linear or non-linear regression or multivariate function estimation in a relatively fast way [28]. It uses a linear set of equations instead of a quadratic programming (QP) problem to obtain the support vectors (SVs). The details of LS-SVM algorithm could be found in the literature [29,30]. The LS-SVM model can be expressed as:

(6) $$y(x)=\sum_{k=1}^{N}\alpha_{k}K(x,x_{k})+b$$

where K(x, x_i_) is the kernel function, xi is the input vector, α_i_ is Lagrange multipliers called support value, b is the bias term.

In the model development using LS-SVM and radial basis function (RBF) kernel, the optimal combination of gam(γ) and sig^2^(σ^2^) parameters was selected when resulting in smaller root mean square error of cross validation (RMSECV). In this study, gam(γ) were optimized in the range of 2^−1^–2^10^ and 2–2^15^ for sig^2^(σ^2^) with adequate increments. These ranges were chosen from previous studies where the magnitude of parameters was optimized. The grid search had two steps the first step was for a crude search with a large step size, and the second step was for the specified search with a small step size. The free LS-SVM toolbox (LS-SVM v 1.5, Suykens, Leuven, Belgium) was applied with MATLAB 7.0 to develop the calibration models.

## 3. Results and Discussion

### 3.1. Overview of Spectra and Statistic Values of Trace Elements

**Figure 1.**
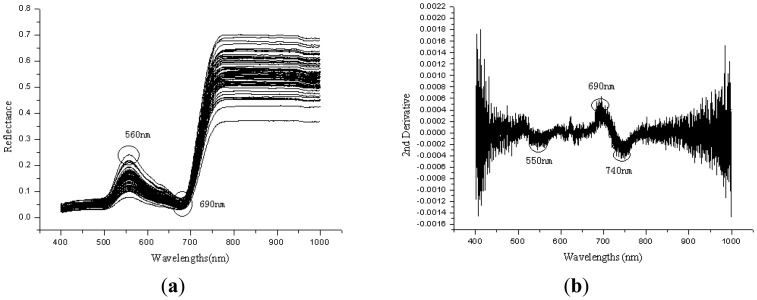
(**a**) The reflectance spectra of all 90 leaf samples in the Vis/NIR region. (**b**) The Vis/NIR spectral curves of leaf samples after 2nd derivative preprocessing.

The lack of trace elements such as Fe, S, Mg, Mn may reduce the chlorophyll content of plant leaf, and will affect the solar radiation absorption by the leaf, so the changes of plant nutritional elements such as nitrogen, water content, and trace elements may directly result in the spectral reflectance changes [31]. Figure 1(a) shows the Vis/NIRS spectral curves of 90 leaf samples. The trend of spectral curves in Vis/NIR region is similar, a small peak appeared at the green band from 560 to 580 nm, and reflectance increased rapidly at about 690–740 nm (red edge) from 10% to 30%–70%. Wavelengths at 580 nm were close to the green pigments, and wavelengths near 680 nm or 710–730 nm was at the red edge position [32].Treated them with 2nd derivative, some peaks and valleys were shown in Figure 1(b). There exists peaks at the wavebands near 690–700 nm, and at the wavebands 720–740 nm and 550–570 nm are troughs.

### 3.2. PLS Models

Calibration models were built between the spectra and content of trace elements (Fe and Zn). Different LVs were applied to build the calibration models, and no outliers were detected in the calibration set during the development of PLS models. The models were used to predict the left 20 samples, and the best performance was achieved with six LVs for Fe and five LVs for Zn. The R^2^, RMSEP and bias were 0.3820, 26.1431 ppm and −9.3674 ppm for Fe, 0.5800, 6.9637 ppm and 2.2320 ppm for Zn, respectively.

### 3.3. LS-SVM Models with Different SWs Selection Methods

#### 3.3.1. PCA-LS-SVM Models

PCs obtained from PCA were applied as inputs of LS-SVM models to improve the training speed and reduce the training error of Vis/NIR model because the training time increased with the square of the number of training samples and linearly with the number of variables. From the aforementioned analysis of the performance of PCA models, the PCs from the Vis/NIR region were used as new eigenvectors to enhance the features of spectra and reduce the dimensionality of the spectra data matrix. Several PCs were extracted from the spectra of 90 samples.

Before the LS-SVM calibration model was built, three steps are crucial for the optimal input feature subset, proper kernel function and the optimal kernel parameters. Firstly, the six PCs obtained from PCA analysis were used as the input data set, and the accumulated contribution of it was reached 95.2%. Secondly, radial basis function could handle the nonlinear relationships between the spectra and target attributes. Finally, two important parameters gam (γ) and sig^2^ (σ^2^) should be optimal for RBF kernel function as aforementioned in multivariate analysis.

**Figure 2.**
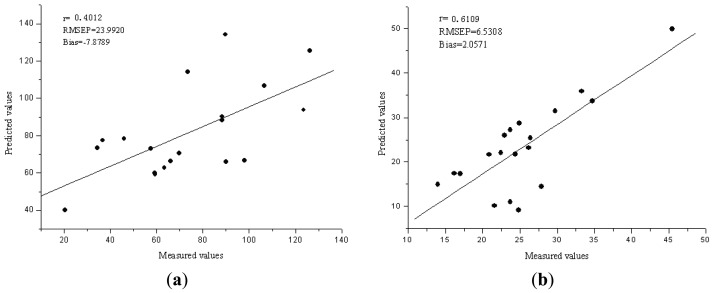
(**a**) The predicted *versus* reference values for Fe by PCA-LS-SVM model. (**b**) The predicted *versus* reference values for Zn by PCA-LS-SVM model.

The performance of the Vis/NIR models was evaluated by 20 samples in validation set. The R^2^, RMSEP and bias for validation sets were 0.4012, 23.9920 ppm and −7.8789 ppm for Fe, 0.6109, 6.5308 ppm and 2.0571 ppm for Zn, respectively. Figure 2(a,b) compare the predicted values and measured values for Fe and Zn, respectively, by the PCA-LS-SVM model. The diagonal line (y = x) shows the ideal results that mean the predicted values are equal to the measured values. The closer the sample plots are to this line, the better is the model. From these figures, the sample plots in the validation sets were distributed near the ideal line for Zn, but the prediction performance is not good for Fe.

#### 3.3.2. LV-LS-SVM Models

Latent variables obtained from PLS were applied as inputs of LS-SVM models to improve the training speed and reduce the training error of Vis/NIR model. From the aforementioned analysis of the performance of PLS models, the LVs from the Vis/NIR region were used as new eigenvectors to enhance the features of spectra and reduce the dimensionality of the spectra data matrix. Several LVs were extracted from the spectra of 90 samples.

**Figure 3.**
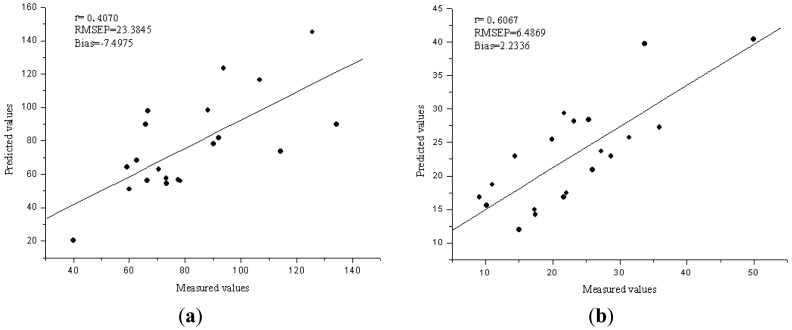
(**a**) The predicted *versus* reference values for Fe by LV-LS-SVM model. (**b**) The predicted *versus* reference values for Zn by LV-LS-SVM model.

The performance of the Vis/NIR models was evaluated by 20 samples in validation set. With a comparison of the results for calibration and validation sets, the best performance was achieved with six LVs for Fe and five LVs for Zn. The R^2^, RMSEP and bias for validation sets were 0.4070, 23.3845 ppm and −7.4975 ppm for Fe, 0.6067, 6.4869 ppm and 2.2336 ppm for Zn, respectively. Figure 3(a,b) show the predicted *versus* reference charts. Compared with the PCA-LS-SVM models, the prediction performance for Fe was improved a little, but still not good. The PCA-LS-SVM calibration model has better performance than the LV-LS-SVM model for Zn.

#### 3.3.3. ICA-LS-SVM Models

**Figure 4.**
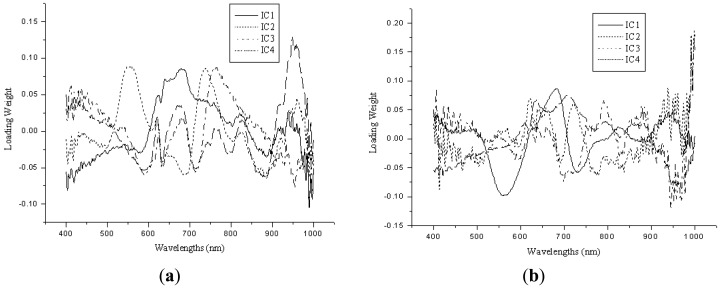
(**a**) The four ICs for Fe by the ICA-LS-SVM model. (**b**) The four ICs for Zn by the ICA-LS-SVM model.

**Figure 5.**
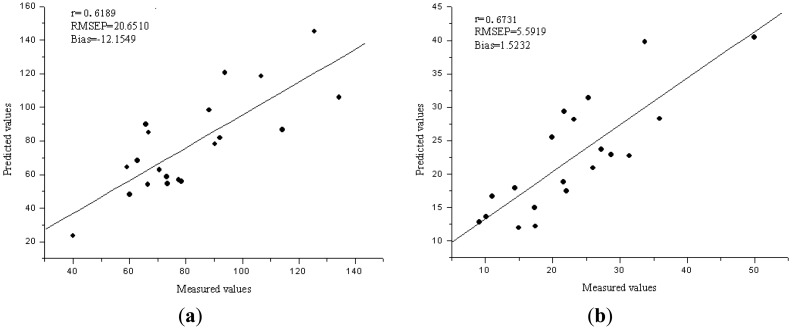
(**a**) The predicted *versus* reference values for Fe by ICA-LS-SVM model. (**b**) The predicted *versus* reference values for Zn by ICA-LS-SVM model.

Independent component analysis was applied for the selection of sensitive wavelengths (SWs), which could reflect the main features of the raw absorbance spectra. FastICA was used to the preprocessed spectra data, and the main absorbance peaks and valleys were indicated by the spectra of ICs. The SWs were selected by the weights of the first four ICs, which wavelengths with the highest weights of each IC were selected as the SWs. Figure 4(a,b) show the four ICs for Fe and Zn. Six SWs were selected corresponding to four ICs, and they were wavelengths near 680, 580, 960, 730, 760 and 830 nm for Fe, 680, 710, 640, 720, 580, and 800 nm for Zn. In order to evaluate the performance of SWs, they were applied as the input data matrix to develop the ICA-LS-SVM models. The validation results showed the R^2^, RMSEP and bias were 0.6189, 20.6510 ppm and −12.1549 ppm for Fe, 0.6731, 5.5919 ppm, 1.5232 ppm for Zn, respectively. Figure 5(a,b) show the predicted *versus* reference graphs. The ICA-LS-SVM models achieved a better performance compared to the best LV-LS-SVM models both in calibration and validation sets. Wavelengths at 580 nm were close to the chlorophyll content of leaf, and wavelengths at 680 nm, 710 nm or 720 nm were near the red edge position. The wavelength 960 nm was close to the water absorbance bands, and it means Fe may affect by the intimidating of water [33]. Therefore, the selection of SWs was suitable for such situation in the present study and the effectiveness of SWs was also validated. The SWs represented most of the features of the original spectra, and could replace the whole wavelength region to predict the trace elements in rice.

Ma *et al*. reported that the element Co had high correlation near the wavelength 569.22 nm, with an R^2^ value of 0.623 [31]. They claimed this might caused by the variation of chlorophyll content. Al Abbas *et al*. studied the spectra of “normal” and six types of nutrient-deficient maize leaves, and it showed that the chlorophyll concentration of leaves in all nutrient-deficiency treatments was lower than of leaves in the control [34]. This was accordant with the results concerning Co in the paper of Ma *et al*. In our study, Fe belongs to the family of iron elements, and Zn is kindred with sulfur elements. Fe and Co belong to the same element family, so it is normal that the spectral response of Fe is similar to that of Co, and both of them have high correlation near the wavelength of 580 nm. For Zn, the sensitive wavelengths near 680, 710 and 720 nm were near the red edge.

### 3.4. Analysis of the Results

Compared with the above PLS, PCA-LS-SVM, LV-LS-SVM and ICA-LS-SVM model, the nonlinear PCA-LS-SVM, LV-LS-SVM, ICA-LS-SVM models turned out to be better than linear model of PLS. The best model was obtained by using the ICA-LS-SVM model for prediction of trace elements in rice. Table 2 shows all the parameters of RMSEP and R^2^ in the four models.

The ICA-LS-SVM models had a better performance, and the reason might be that the LS-SVM models took the nonlinear information of the spectral data into consideration and the nonlinear information had improved the prediction precision. The ICs from ICA were obtained by a high-order statistic that is much stronger condition than orthogonality, so the SWs selected from ICs were more effective, and it could be very helpful for the development of portable instrument or real-time monitoring of the rice trace elements.

## 4. Conclusions

Vis/NIR spectroscopy was successfully utilized for the determination of some trace elements (Fe, Zn) in rice. A new combination of ICA-LS-SVM was proposed with comparison of nonlinear LV-LS-SVM models, PCA-LS-SVM modes and linear PLS models. ICA-LS-SVM model turned out to be the best for prediction of trace elements in rice, and was better than the nonlinear LV-LS-SVM model. The R^2^, RMSEP and bias by ICA-LS-SVM were 0.6189, 20.6510 ppm and −12.1549 ppm for Fe, and 0.6731, 5.5919 ppm and 1.5232 ppm for Zn, respectively. The overall results demonstrated ICA was a powerful tool for variable selection, and the newly proposed ICA-LS-SVM method could be applied as an alternative fast and accurate method for the determination of trace elements in rice.

## Figures and Tables

**Table 1.** The statistic values of Fe and Zn contents in calibration and validation sets.

**Element**	**Data set**	**Samples**	**Range (ppm)**	**Mean (ppm)**	**Standard deviation (ppm)**
Fe	Calibration	70	39.951–134.254	79.245	21.945
	Validation	20	40.011–133.992	80.649	24.003
	All samples	90	39.951–134.254	80.935	23.669
Zn	Calibration	70	9.085–49.927	23.710	9.221
	Validation	20	9.103–49.849	23.134	9.518
	All samples	90	9.085–49.927	23.408	9.928

**Table 2.** The parameters of RMSEP and R^2^ in the four models.

**Models**	**Element**	**Variables No.**	**R^2^**	**RMSEP**	**Bias**
PLS	Fe	6	0.3820	26.1431	−9.3674
	Zn	5	0.5800	6.9637	2.2320
PCA-LS-SVM	Fe	6	0.4012	23.9920	−7.8789
	Zn	6	0.6109	6.5308	2.0571
LV-LS-SVM	Fe	6	0.4070	23.3845	−7.4975
	Zn	5	0.6067	6.4869	2.2336
ICA-LS-SVM	Fe	6	0.6189	20.6510	−12.1549
	Zn	6	0.6731	5.5919	1.5232
